# Rabies Vaccination Compliance and Reasons for Incompletion

**DOI:** 10.5811/westjem.2020.3.45893

**Published:** 2020-07-02

**Authors:** Tony Shi, Eleanor F. Dunham, Jennifer E. Nyland

**Affiliations:** * The Pennsylvania State University College of Medicine, Hershey, Pennsylvania; † Penn State Milton S. Hershey Medical Center, Department of Emergency Medicine, Hershey, Pennsylvania; ‡ The Pennsylvania State University College of Medicine, Department of Neural and Behavioral Sciences, Hershey, Pennsylvania

## Abstract

**Introduction:**

Rabies is a fatal disease with a 91% mortality rate in the United States. Current treatment of rabies consists of post-exposure prophylaxis treatment involving a complicated vaccination regimen. Studies conducted in other countries have found that patients do not complete their rabies vaccination treatment due to forgetting about their treatment, lack of time for visits, and the financial burden of treatment. However, little is known about why patients do not complete the rabies series in the US. The objective of this study was to determine the reasons why patients in the US do not complete rabies treatment.

**Methods:**

We performed a retrospective study to evaluate rabies post-exposure prophylaxis completion in the emergency department of an academic suburban hospital between June 2014– July 2017. Further review was performed for patients who received inadequate vaccination to determine the cause of treatment incompletion. We conducted additional follow-up by phone survey for those patients who did not complete their rabies treatment but had no explanation for discontinuation available in the medical chart review.

**Results:**

Results indicated 198 patients received rabies post-exposure treatment during the inclusion period. Of these, 145 patients completed the rabies vaccination regimen. Reasons for treatment incompletion were found for 29 patients, and 24 patients were lost to follow-up. Of the 29 patients for which discontinuation was assessed, 23 patients (79.3%) stopped treatment due to appropriate reasons – either the animal involved tested negative for the rabies virus or the patient had prior rabies treatment and only required two booster shots. Reasons for not completing the series when medically indicated included the patient deciding to not return for treatment, lack of awareness of the full vaccination regimen, and the patient declining initiation of rabies vaccination.

**Conclusion:**

Most patients in the US discontinue their rabies vaccination treatment for appropriate reasons; however, there is a proportion of patients who discontinue rabies vaccination when further treatment is medically indicated. This subset of patients is particularly at risk of rabies-related mortality, and additional measures need to be taken to ensure increased treatment compliance.

## INTRODUCTION

Globally, there are an annual estimated 59,000 deaths due to rabies,^1^ most of which are attributed to domesticated dogs. Fortunately in the United States, the prevalence of rabies in domestic animals has been drastically reduced due to mandatory vaccination of pets. However; the Centers for Disease Control and Prevention (CDC) still report 1–3 rabies cases each year in the US despite 30,000–60,000 prophylaxis treatments given annually.^1^ This is because many variants of the rabies virus can still be found in various wildlife, particularly among bats and raccoons.^5^ According to the *Pennsylvania Animal Rabies 2017 Report*, thousands of animal are tested for rabies annually in Pennsylvania and on average 392 animals per year have tested positive in the past 10 years.^6^ Despite the precautions taken, 23 cases of rabies in humans were reported to the CDC between 2008–2017, resulting in 21 deaths.^1^ These statistics show that there is still a risk of rabies infection for patients in the US, especially during the warmer months due to increased encounters with wildlife.

The current standard of care for rabies is administration of the rabies vaccination after potential exposure. The current rabies post-exposure treatment regimen in the US is one dose of human rabies immune globulin plus one dose of the rabies vaccine immediately after the exposure. Three additional doses of the rabies vaccine are administered three, seven, and 14 days after the initial dose.^7^ Unfortunately, this is a complicated regimen and previous investigations within our emergency department (ED) have revealed that some patients do not return to complete the recommended treatment.

Multiple studies have been performed in other countries, including China and Thailand, evaluating the reasons why patients do not complete their rabies treatment. Some results reported patients forgetting about their treatment, lack of time for visits, and the financial burden of treatment.^2^,^3^,^4^ However, no studies have been conducted in the US to evaluate causes for failure to complete the rabies vaccination regimen. Our objective was to determine the reasons why patients in the US discontinue rabies vaccination treatment. The results of this study can be used to help formulate countermeasures for increasing treatment compliance and reduce the risk of fatalities.

## METHODS

This study was a single-center, retrospective cohort analysis designed to assess the completion rate and reason for discontinuation of post-exposure rabies treatment in patients seen in the ED. The study was performed in an academic, suburban hospital ED with an annual census of 70,000 visits per year. All procedures for this study were approved by the institutional review board. Training was provided to the primary author (TS) to review charts and collect data using a standardized abstraction form. The research team met frequently to discuss discrepancies and maintain consistency in data abstracted.

We obtained patient data from the Enterprise Information Management (EIM) office. The EIM is tasked with structuring and governing information across the organization, management of health records, and acquisition of patient charts for quality improvement and clinical research. The data obtained from EIM included patients of all ages who initiated rabies post-exposure treatment in the ED between June 2014–July 2017. We conducted an additional search for all animal bites seen in the ED within the same timeframe. These patients were cross-matched with patients who initiated rabies post-exposure prophylaxis treatment. The results show that all patients seen for an animal bite were offered post-exposure prophylaxis treatment.

**Population Health Research Capsule**

What do we already know about this issue?

*Rabies is a preventable viral disease when treated with post-exposure prophylaxis vaccination. Some patients do not complete treatment and are at risk of rabies-related mortality*.

What was the research question?

What are the reasons patients do not complete post-exposure prophylaxis treatment?

What was the major finding of the study?

*While the majority of patients appropriately stopped treatment, a few discontinued treatment when not indicated*.

How does this improve population health?

*Patients inappropriately discontinue vaccination due to inadequate knowledge of treatment course and illness severity. Increased education and follow-up care is needed for these patients*.

Records identified by the EIM then underwent chart review. The chart review involved utilization of each patient’s “immunization history” section and the “chart search” functionality of the electronic health record. The search term “rabies” was used to bring up all notes mentioning the term, and each individual note was reviewed to evaluate completion of rabies treatment. Patients who received the four CDC recommended doses were considered to have completed treatment and were included in the “completed vaccination” group without further analysis.

We conducted a retrospective chart review for patients without record of treatment completion in the immunization history. The initial post-exposure encounter note was evaluated to determine the animal involved in the exposure and initial treatment. Medical notes for later encounters were reviewed to evaluate reasons why patients did not return for completion of their rabies vaccine treatment and the number of vaccine doses each patient received prior to termination of treatment. If an explanation was provided for incompletion of treatment, the chart review was considered to be complete for that patient and the reason for discontinuation was recorded.

If a reason for incompletion was not found through chart review, the patient was subsequently contacted via phone. A phone script was followed to determine the reason why patients did not complete the rabies vaccine treatment. Multiple attempts to contact each patient were made, altering days and times to maximize responses; patients were considered lost to follow-up if they did not answer their phone after 10 attempts. Verbal consent for inclusion in a research study was obtained from each subject according to the phone script. For underage subjects, verbal consent was obtained from parents and/or guardians. Subjects who were unable/unwilling to provide consent or unable to speak English were considered lost to follow-up. Patients who confirmed receipt of the four recommended doses were included in the “completed vaccination” group.

Patients who did not complete treatment were categorized into two groups: patients whose treatment termination was medically indicated (ie, animal involved tested negative for rabies or patient had received previous vaccination and only required two booster shots), and those whose treatment termination was not medically indicated.

## RESULTS

A total of 198 patients received rabies treatment between June 2014–July 2017. The study population was representative of the larger patient population seen in this ED (Table 1). Of these patients, 145 (73.2%) completed treatment, 29 (14.6%) had incomplete treatment, and 24 (12.1%) were lost to follow-up (Figure).

Of those patients who discontinued treatment early, the majority (79.3%) were found to have terminated their treatments appropriately (Table 2). The remaining patients (20.7%) terminated rabies treatment when it was not medically indicated. Results show that dogs were involved with the majority of incidents with bats as the second most frequently associated animal (Table 3). Of the six patients who stopped treatment when it was not medically indicated, three cases involved dogs, two cases involved bats, and one case involved a deer. Results demonstrate that the majority of patients (65.5%) who discontinued treatment received only one dose of the vaccine prior to discontinuation (Table 4). A second investigator reviewed 20 charts selected at random. The percent agreement between the two abstractors was 90% for treatment completion.

## DISCUSSION

Rabies is a fatal disease that is easily preventable when appropriately treated with post-exposure prophylaxis. However, a previous investigation at our institution demonstrated that many patients seen in our ED for rabies post-exposure prophylaxis do not complete the recommended treatment course. Multiple studies have reported reasons why patients do not return to complete their rabies vaccination treatments, but all of these have been performed in developing countries where resources are limited. There has not yet been a study dedicated to evaluation of patients in the US.

This study specifically examined the reasons why patients at a single, academic institution in the US discontinue rabies vaccination treatment. Results show that the majority of patients do not return for completion of the vaccination regimen due to appropriate reasons (ie, the animal involved tested negative for the rabies virus or the patient had completed prior rabies treatment and only required two booster shots). This varied from prior studies performed in other countries (eg, Thailand and China), in which the main reasons for rabies vaccination incompletion were patients not remembering their treatment, lack of time for visits, and the financial burden of treatment.^2^,^3^,^4^

This difference is likely due to higher accessibility of resources available to patients in the US, particularly the opportunity to have the involved animals tested to determine the need for further medical treatment. This is especially noteworthy in the state of Pennsylvania where in-state residents are offered free animal testing, providing an opportunity for patients to avoid the financial cost and physical discomfort associated with rabies vaccinations.^8^ Additionally, the extensive treatment course allows ample time to obtain animal testing results before further vaccination treatment is indicated. This was highlighted by the fact that the majority of patients who stopped treatment only received one dose of the vaccine.

Additionally, our results show that dogs were involved with the majority of the incompletion cases. Cats and dogs are considered low risk and less likely to transmit rabies to humans due to mandatory vaccination of domesticated animals.^7^ However, patients seen in our ED were offered treatment as a precaution because of unknown vaccination status of these animals. Furthermore, there is a small chance of transmission given that 50–60 dogs and over 250 cats test positive for rabies in the US each year, many of which were unvaccinated and infected by wildlife.^1^ The animals were available for testing in some of these cases and were more likely to test negative given their lower risk of infection, thereby increasing the chances of appropriate treatment termination.

Nevertheless, there is still a proportion of patients who stopped vaccination treatment when further treatment was indicated. Additionally, it is reasonable to infer that some patients who were lost to follow-up did not receive the full vaccination regimen when medically indicated. Even though these patients only represent a small proportion of all patients receiving rabies treatment, this specific subset of patients is particularly at risk of rabies-related mortality despite rabies being an illness that is easily preventable with the right interventions. Therefore, it is important that additional steps be taken to increase treatment compliance.

Patient education and close follow-up are integral steps to increasing vaccination treatment compliance. With the prevalence of technology in everyday life, we propose the potential of electronic messaging to help increase vaccination completion rates. In many institutions, patients can sign up for patient portals that allow electronic communication between patients and medical providers. This provides a platform to educate patients about rabies and the potential harms of inadequate treatment. Additionally, previous studies have shown the utility of text messaging for increasing patient follow-up after ED visits.^9^ Therefore, these are two approaches that can facilitate communication between patients and providers to improve treatment compliance.

Our results show the average age of patients to be 34.3 years old among the completed vaccination group and 29.9 years old among the incomplete vaccination group. These values highlight the prevalence of younger patients being treated for post-exposure rabies treatment. The age distinction is hypothesized to be representative of the population that is more likely to encounter unfamiliar animals. Younger individuals spend more time outdoors and are more likely to encounter wildlife, leading to higher chances of exposure. When separated by age, treatment completion rates were similar for younger patients (93.2% among young adults) and older patients (85.7% among older adults). Nevertheless, additional studies can stratify patients by completion rate among different patient age groups to identify patients who are at a higher risk of non-compliance with post-exposure treatment.

## LIMITATIONS

There are multiple factors in this study that limit the generalizability of the results. The main limiting factor is that this study is a single-center study. It was conducted in a rural/suburban area with a predominantly Caucasian population; therefore, our findings may not be generalizable to other parts of the country where the demographics are different. Additionally, reasons for inappropriate incompletion of treatment were determined for only six patients. Due to such a small sample size, no distinct conclusions can be made for recommendation of change. Lastly, a large number of patients were lost to follow-up, which could have potentially skewed the final results of the study. We postulate that the patients lost to follow-up reflect the results obtained from the study, given that three of these patients had the animal involved tested or quarantined and many other patients could have completed their treatment elsewhere. However, these are not certain and therefore limit the final results of our study.

No blinding was involved in data abstraction because chart reviews were performed by the primary investigator. Patient consent for communication other than via phone survey was unable to be obtained due to the retrospective nature of this study. Therefore, no additional measures were taken to contact patients, leading to high number of patients lost to follow-up.

## CONCLUSION

Results show that the majority of patients received the appropriate post-exposure treatment (ie, completed the vaccination regimen or stopped treatment when medically indicated). However, a proportion of patients was found to have stopped their rabies treatment prior to completion and at increased risk of rabies-related mortality. This demonstrates that current medical practice leads to proper rabies management for the majority of patients, but there is a small subset of patients who do not complete their vaccination regimen and are at higher risk of rabies-related mortality. Therefore, additional measures need to be taken to ensure increased treatment compliance, mainly in the form of patient education to increase awareness of the high mortality associated with improper treatment.

## Figures and Tables

**Figure:**
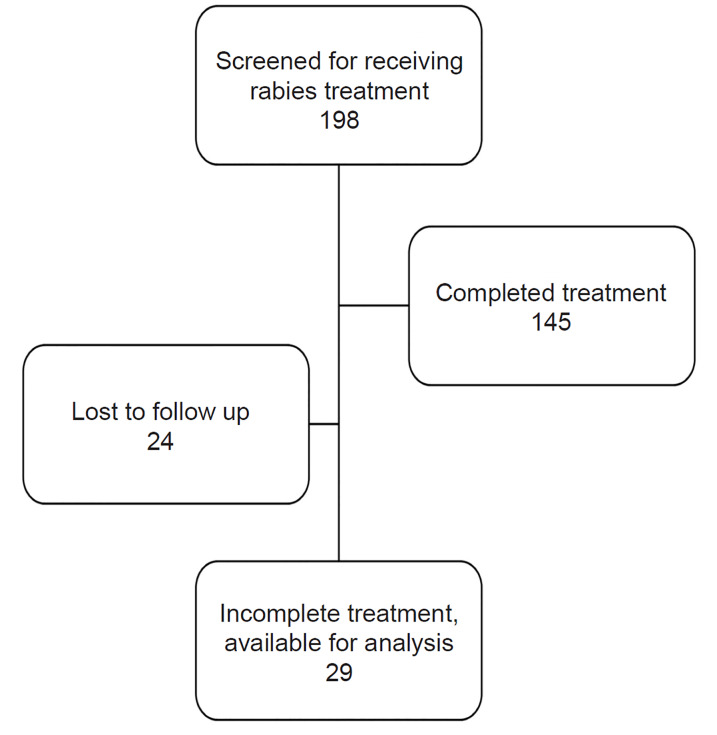
Treatment outcome grouping of patients who did or did not complete rabies vaccination regimen.

**Table 1:** Treatment outcome and patient demographics of patients exposed to possible rabies infection.

Feature	Completed vaccination	Incomplete vaccination	Lost to follow up
Total patients	145 (73.2%)	29 (14.6%)	24 (12.1%)
Age group			
Pediatric	35 (24.1%)	12 (41.4%)	11 (45.8%)
Young adults	48 (33.1%)	4 (13.8%)	6 (25.0%)
Middle-aged adults	38 (26.2%)	9 (31.0%)	5 (20.8%)
Older adults	24 (16.6%)	4 (13.8%)	2 (8.3%)
Average age	34.3	29.9	26.2
Gender			
Male	67 (46.2%)	8 (27.6%)	12 (50.0%)
Female	78 (53.8%)	21 (72.4%)	12 (50.0%)
Race			
Caucasian	136 (93.8%)	25 (86.2%)	19 (79.2%)
African American	2 (1.4%)	2 (6.9%)	0 (0.0%)
Hispanic	5 (3.4%)	1 (3.4%)	5 (20.8%)
Asian	0 (0.0%)	1 (3.4%)	0 (0.0%)
Other	2 (1.4%)	0 (0.0%)	0 (0.0%)

Pediatric: < 18; young adults: 18–35; middle-aged adults: 36–55; older adults: >55.

**Table 2:** Reasons for incompletion of vaccination regimen.

Reasons for incomplete	n (%)
Animal tested negative	18 (62.1%)
Received prior vaccination, only require booster shots	5 (17.2%)
Patient decided not to return to complete vaccination	3 (10.3%)
Patient unaware of vaccination regimen	2 (6.9%)
Patient declined initial vaccination	1 (3.4%)
Total	29

**Table 3:** Animals involved in rabies bites.

Animals involved	n (%)
Dog*	18 (62.1%)
Bat*	7 (24.1%)
Deer*	2 (6.9%)
Cat	1 (3.4%)
Raccoon	1 (3.4%)

* Animals involved in cases where patients terminated treatment when not medically indicated.

**Table 4:** Number of vaccines received by patients with suspected rabies exposure.

Number of vaccines	n (%)
Zero	1 (3.4%)
One	19 (65.5%)
Two	5 (17.2%)
Three	4 (13.8%)
